# HTS explorer

**DOI:** 10.1186/1758-2946-6-S1-P20

**Published:** 2014-03-11

**Authors:** Christoph Müller, Isabella Feierberg, Ola Engkvist, Christian Tyrchan

**Affiliations:** 1 AstraZeneca R&D, Discovery Sciences Chemistry Innovation Centre & RIA iMed Medicinal Chemistry, Mölndal, Sweden

## 

In the early stages of a drug discovery project it is often necessary to narrow down the search space for potential new leads substantially [1,2]. This crucial step identifies a set of molecules (a hit series) that have a high likelihood of being relevant to the drug discovery project. In many cases high throughput screening (HTS) is used to test (in-vitro) large amounts of molecules against a biological target in order to validate a molecule’s potential to interact with the target and therewith its relevance to the drug discovery process. Since there are time and money constraints associated with such a process, it is not feasible to pipe the full HTS compound set through very detailed testing. Rather, an HTS process consists of several stages: a primary (spot test, SP) stage, a confirmation (retest) stage and a concentration-response (CR) stage. The later is the most resource-intensive where compounds are tested at a range of different concentrations, which allows for curve-fitting and determination of the potencies.

The HTS Explorer enables HTS evaluators across AstraZeneca R&D sites to do a comprehensive and effective screening analysis for compound prioritization within a single tool. This tool is provided as an extension to the visualization platform TIBCO Spotfire [3]. The use of Spotfire as a platform streamlines the process for the HTS evaluator and facilitates distribution of the tool as sharing of the HTS data evaluations. Further new approaches and methods can be made easily and readily available across AZ. Data is retrieved by calling PipelinePilot protocols, various web-services and queries to in-house databases [4,5]. The main features include many different options for clustering of compounds as well as commenting on clusters, cluster visualization, prioritization and interactive reclustering. Further it enables compound annotation with e.g. the known Structure-Activity Relationship (SAR) space and offers different types of structural searches in internal and external databases.
